# Supplementary material for: {GdIII7} and {GdIII14} Cluster Formation Based on a Rhodamine 6G Ligand with a Magnetocaloric Effect
Source: Molecules. 2024 Jan 12;29(2):389. doi: 10.3390/molecules29020389 (PMC10820868; doi:10.3390/molecules29020389)
Supplement: Supplementary file 1 [file molecules-29-00389-s001.zip › molecules-2826122-supplementary.pdf]

Supporting Information

{Gd^{III}_7} and {Gd^{III}_{14}} clusters based on Rhodamine 6G ligand with magnetocaloric effect

Lin Miao [1], Cai-Ming Liu [2] and Hui-Zhong Kou [1,*]

1 Department of Chemistry, Tsinghua University, Beijing 100084, P. R. China.
2 Beijing National Laboratory for Molecular Sciences, Center for Molecular Science, Institute of Chemistry, Chinese Academy of Sciences, Beijing 100190, P. R. China.
* Correspondence: kouhz@mail.tsinghua.edu.cn (H.-Z.K.)

Table S1. Crystal data and refinement parameters for complexes **1** and **2**.

Complex	**1**	**2**
Formula	$C_{236}H_{288}N_{37}O_{51}Gd_7$	$C_{285}H_{330.5}N_{57.5}O_{104}Gd_{14}$
Formula weight	5559.77	8427.05
T / K	100	173
Crystal system	monoclinic	tetragonal
Space group	$P2_1$/n	$P4$/n
a / Å	26.4695(2)	27.7525(9)
b / Å	57.9994(6)	27.7525(9)
c / Å	52.5818(6)	24.9438(7)
α / º	90	90
β / º	98.2830(10)	90
γ / º	90	90
V / Å^3	79882.3(14)	19211.7(13)
Z	12	2
ρ_{calc} / g·cm^{-3}	1.387	1.457
μ / mm^{-1}	11.692	16.005
F(000)	33828	8342
Radiation(CuKα)	CuKα (λ = 1.54184 Å)	CuKα (λ = 1.54184 Å)
Data/restraints/parameters	133115/66/7410	19020/78/1042
GOF on F^2	1.073	0.981
R_{int}	0.0759	0.0921
$R1$ [$I > 2\sigma(I)$]	0.1210	0.0900
$R1$,$wR2$(all data)	0.3341	0.3012
CCDC	2322492	2322493

Table S2. Selected bond distances (Å) and bond angles (°) for complex **1**.

Gd1-O1 (μ_3-OH^-)	2.340(9)	Gd1-O5 (μ_3-CH_3O^-)	2.362(9)
Gd1-O2 (μ_3-OH^-)	2.358(9)	Gd1-O6 (μ_3-CH_3O^-)	2.387(9)
Gd1-O3 (μ_3-OH^-)	2.338(9)	Gd1-O7 (μ_3-CH_3O^-)	2.393(9)
Gd1-O4 (μ_3-OH^-)	2.316(9)	Gd1-O8 (μ_3-CH_3O^-)	2.404(6)
Gd2-O3 (μ_3-OH^-)	2.375(8)	Gd2-O15 (NO_3^-)	2.503(11)
Gd2-O5 (μ_3-CH_3O^-)	2.311(9)	Gd2-O19 (μ_2-phO$^-$)	2.342(9)
Gd2-O6 (μ_3-CH_3O^-)	2.461(9)	Gd2-O20 (acyl oxygen)	2.463(10)
Gd2-O11 (μ_2-CH_3O^-)	2.312(9)	Gd2-N3	2.615(13)
Gd2-O13 (NO_3^-)	2.485(10)		
Gd3-O5 (μ_3-CH_3O^-)	2.474(9)	Gd3-O19 (μ_2-phO$^-$)	2.389(9)
Gd3-O6 (μ_3-CH_3O^-)	2.314(9)	Gd3-O22 (phO$^-$)	2.229(9)
Gd3-O10 (μ_2-CH_3O^-)	2.298(10)	Gd3-O23 (acyl oxygen)	2.412(9)
Gd3-N7	2.536(11)	Gd3-O1 (μ_3-OH^-)	2.496(9)
Gd4-O9 (μ_2-CH_3O^-)	2.288(10)	Gd4-O25 (phO$^-$)	2.189(10)
Gd4-O10 (μ_2-CH_3O^-)	2.261(10)	Gd4-O2 (μ_3-OH^-)	2.331(9)
Gd4-O26 (acyl oxygen)	2.390(10)	Gd4-N11	2.585(12)
Gd4-O1 (μ_3-OH^-)	2.315(9)		
Gd5-O2 (μ_3-OH^-)	2.384(9)	Gd5-O16 (NO_3^-)	2.533(11)
Gd5-O8 (μ_3-CH_3O^-)	2.328(6)	Gd5-O18 (NO_3^-)	2.470(12)
Gd5-O9 (μ_2-CH_3O^-)	2.298(10)	Gd5-O29 (μ_2-phO$^-$)	2.342(9)
Gd5-O7 (μ_3-CH_3O^-)	2.507(9)	Gd5-O28 (acyl oxygen)	2.418(10)
Gd5-N15	2.653(12)		
Gd6-O7 (μ_3-CH_3O^-)	2.314(9)	Gd6-O31 (acyl oxygen)	2.380(10)
Gd6-O8 (μ_3-CH_3O^-)	2.464(6)	Gd6-O32 (phO$^-$)	2.218(11)
Gd6-O12 (μ_2-CH_3O^-)	2.283(10)	Gd6-N19	2.511(12)
Gd6-O29 (μ_2-phO$^-$)	2.433(9)	Gd6-O4 (μ_3-OH^-)	2.505(9)
Gd7-O4 (μ_3-OH^-)	2.302(9)	Gd7-O34 (phO$^-$)	2.257(10)
Gd7-O11 (μ_2-CH_3O^-)	2.267(9)	Gd7-O35 (acyl oxygen)	2.398(10)
Gd7-O12 (μ_2-CH_3O^-)	2.261(10)	Gd7-N23	2.564(11)
Gd7-O3 (μ_3-OH^-)	2.336(8)		
Gd1---Gd2	3.5577(8)	Gd1---Gd3	3.5064(11)
Gd1---Gd5	3.5583(11)	Gd1---Gd6	3.5088(10)
Gd1---Gd4	3.808(11)	Gd1---Gd7	3.797(11)
Gd2---Gd3	3.5824(9)	Gd6---Gd7	3.7832(7)
Gd3---Gd4	3.7955(11)	Gd4---Gd5	3.7699(12)

Gd5---Gd6	3.611(12)	Gd2---Gd7	3.760(11)
Gd1-O2-Gd5	97.3(3)	Gd4-O2-Gd1	108.6(4)
Gd4-O2-Gd5	106.2(4)	Gd1-O1-Gd3	92.9(3)
Gd4-O1-Gd1	109.7(3)	Gd4-O1-Gd3	104.1(3)
Gd1-O3-Gd2	98.0(3)	Gd7-O3-Gd1	108.6(3)
Gd7-O3-Gd2	105.9(3)	Gd7-O4-Gd6	103.7(3)
Gd1-O4-Gd6	93.3(3)	Gd7-O4-Gd1	110.6(4)
Gd1-O5-Gd3	92.9(3)	Gd1-O7-Gd5	93.1(3)
Gd2-O5-Gd1	99.2(3)	Gd1-O6-Gd2	94.4(3)
Gd6-O7-Gd1	96.4(3)	Gd2-O5-Gd3	96.9(3)
Gd3-O6-Gd1	96.5(3)	Gd6-O7-Gd5	96.9(3)
Gd5-O8-Gd6	97.7(2)	Gd3-O6-Gd2	97.2(3)
Gd1-O8-Gd6	92.2(2)	Gd5-O8-Gd1	97.5(2)
Gd7-O11-Gd2	110.4(4)	Gd7-O12-Gd6	112.8(4)
Gd4-O9-Gd5	110.6(4)	Gd4-O10-Gd3	112.8(4)
Gd5-O29-Gd6	98.2(3)	Gd2-O19-Gd3	98.4(3)
O6-Gd3-N7	150.5(3)	O2-Gd4-N11	136.5(3)
O23-Gd3-O1	70.9(3)	O2-Gd4-O26	74.1(3)
O23-Gd3-O5	84.2(3)	O1-Gd4-O2	70.8(3)
O23-Gd3-N7	65.2(3)	O1-Gd4-N11	152.7(3)
O10-Gd3-O1	68.9(3)	O1-Gd4-O26	139.3(3)
O10-Gd3-O5	132.3(3)	O9-Gd4-O2	72.1(3)
O10-Gd3-O19	158.4(3)	O9-Gd4-O1	101.2(3)
O10-Gd3-O6	97.7(3)	O9-Gd4-N11	91.2(4)
O10-Gd3-O23	95.5(3)	O9-Gd4-O26	86.8(3)
O10-Gd3-N7	85.8(4)	O10-Gd4-O2	92.4(3)
O2-Gd5-N2	121.6(4)	O7-Gd5-N2	158.3(4)
O2-Gd5-N15	127.7(3)	O7-Gd5-N15	87.0(3)
O2-Gd5-O7	70.6(3)	O7-Gd5-O16	144.7(3)
O2-Gd5-O28	65.4(3)	O8-Gd5-O2	76.9(3)
O2-Gd5-O16	101.0(3)	O8-Gd5-N2	135.3(3)
O2-Gd5-O18	141.3(4)	O8-Gd5-N15	132.7(3)
N15-Gd5-N2	71.4(4)	O8-Gd5-O7	61.9(3)
O2-Gd1-O5	126.3(3)	O8-Gd5-O29	68.7(3)
O2-Gd1-O7	73.1(3)	O8-Gd5-O28	131.2(3)
O2-Gd1-O8	75.9(3)	O8-Gd5-O16	151.7(3)
O2-Gd1-O6	79.0(3)	O8-Gd5-O18	115.5(3)
O1-Gd1-O2	69.9(3)	O29-Gd5-O2	140.5(3)
O1-Gd1-O5	70.3(3)	O29-Gd5-N2	96.8(4)
O1-Gd1-O7	136.5(3)	O29-Gd5-N15	69.9(3)
O1-Gd1-O8	86.7(3)	O29-Gd5-O7	76.3(3)
O1-Gd1-O6	83.3(3)	O29-Gd5-O28	126.7(3)
O3-Gd1-O2	128.3(3)	O29-Gd5-O16	118.5(3)

O3-Gd1-O1	143.8(3)	O29-Gd5-O18	73.8(4)
O3-Gd1-O5	74.6(3)	O9-Gd5-O2	71.0(3)
O3-Gd1-O7	78.4(3)	O9-Gd5-N2	71.6(4)
O3-Gd1-O8	125.6(3)	O9-Gd5-N15	142.8(3)
O3-Gd1-O6	72.4(3)	O9-Gd5-O7	129.8(3)
O5-Gd1-O7	153.0(3)	O9-Gd5-O8	78.9(3)
O5-Gd1-O8	135.0(3)	O9-Gd5-O29	118.7(3)
O5-Gd1-O6	61.9(3)	O9-Gd5-O28	114.0(3)
O4-Gd1-O2	144.5(3)	O9-Gd5-O16	73.9(4)
O4-Gd1-O1	115.5(3)	O9-Gd5-O18	75.7(4)
O4-Gd1-O3	69.7(3)	O28-Gd5-N2	92.0(4)
O4-Gd1-O5	85.7(3)	O28-Gd5-N15	63.7(3)
O4-Gd1-O7	83.4(3)	O28-Gd5-O7	76.7(3)
O4-Gd1-O8	69.6(3)	O28-Gd5-O16	68.9(4)
O4-Gd1-O6	135.3(3)	O28-Gd5-O18	113.3(4)
O7-Gd1-O8	62.5(3)	O16-Gd5-N2	25.2(4)
O6-Gd1-O7	111.2(3)	O16-Gd5-N15	71.0(4)
O6-Gd1-O8	154.9(3)	O18-Gd5-N2	25.0(4)
O3-Gd2-O6	70.4(3)	O18-Gd5-N15	72.6(4)
O3-Gd2-O20	66.9(3)	O18-Gd5-O7	148.1(4)
O3-Gd2-O15	100.0(3)	O18-Gd5-O16	50.1(4)
O3-Gd2-O13	142.0(3)	O4-Gd6-N19	126.0(3)
O3-Gd2-N3	128.8(3)	O7-Gd6-N19	150.8(4)
O3-Gd2-N1	119.9(4)	O7-Gd6-O4	81.0(3)
O5-Gd2-O3	74.9(3)	O7-Gd6-O31	143.5(3)
O5-Gd2-O11	78.3(3)	O7-Gd6-O8	62.7(3)
O5-Gd2-O19	68.1(3)	O7-Gd6-O29	78.3(3)
O5-Gd2-O6	61.5(3)	O31-Gd6-N19	63.9(4)
O5-Gd2-O20	130.3(3)	O31-Gd6-O4	73.3(3)
O5-Gd2-O15	152.2(3)	O31-Gd6-O8	83.0(3)
O5-Gd2-O13	116.2(3)	O31-Gd6-O29	99.4(3)
O5-Gd2-N3	132.2(4)	O8-Gd6-N19	134.1(3)
O5-Gd2-N1	137.6(4)	O8-Gd6-O4	65.7(2)
O11-Gd2-O3	71.0(3)	O29-Gd6-N19	88.7(3)
O11-Gd2-O19	115.9(3)	O29-Gd6-O4	130.8(3)
O11-Gd2-O6	129.8(3)	O29-Gd6-O8	65.1(2)
O11-Gd2-O20	115.5(3)	O12-Gd6-N19	83.8(4)
O11-Gd2-O15	74.3(3)	O12-Gd6-O4	68.9(3)
O11-Gd2-O13	75.8(3)	O12-Gd6-O7	98.6(3)
O11-Gd2-N3	143.8(4)	O12-Gd6-O31	95.8(3)
O11-Gd2-N1	71.4(4)	O12-Gd6-O8	132.9(3)
O19-Gd2-O3	139.2(3)	O12-Gd6-O29	158.0(3)
O19-Gd2-O6	77.1(3)	O32-Gd6-N19	70.8(4)
O19-Gd2-O20	128.1(3)	O32-Gd6-O4	141.3(4)

O19-Gd2-O15	120.8(3)	O32-Gd6-O7	80.9(4)
O19-Gd2-O13	72.9(3)	O32-Gd6-O31	134.7(4)
O19-Gd2-N3	71.1(4)	O32-Gd6-O8	131.7(3)
O19-Gd2-N1	99.3(4)	O32-Gd6-O29	77.7(4)
O6-Gd2-O20	76.3(3)	O32-Gd6-O12	80.3(4)
O6-Gd2-O15	143.4(3)	O3-Gd7-O35	72.4(3)
O6-Gd2-O13	147.5(3)	O3-Gd7-N23	134.0(3)
O6-Gd2-N3	86.1(3)	O4-Gd7-O3	70.0(3)
O6-Gd2-N1	158.1(4)	O4-Gd7-O35	137.2(3)
O20-Gd2-O15	67.7(3)	O4-Gd7-N23	155.9(3)
O20-Gd2-O13	113.5(4)	O11-Gd7-O3	72.5(3)
O20-Gd2-N3	63.4(4)	O11-Gd7-O4	102.3(3)
O20-Gd2-N1	89.9(4)	O11-Gd7-O35	84.9(3)
O15-Gd2-N3	72.5(4)	O11-Gd7-N23	86.3(3)
O15-Gd2-N1	24.5(4)	O34-Gd7-O3	149.2(3)
O13-Gd2-O15	52.4(4)	O34-Gd7-O4	84.9(3)
O13-Gd2-N3	72.8(4)	O34-Gd7-O11	96.8(3)
O13-Gd2-N1	28.0(4)	O34-Gd7-O12	96.6(4)
N3-Gd2-N1	72.4(4)	O34-Gd7-O35	136.8(3)
O1-Gd3-N7	126.3(3)	O34-Gd7-N23	71.7(4)
O5-Gd3-O1	66.0(3)	O12-Gd7-O3	92.8(3)
O5-Gd3-N7	134.3(3)	O12-Gd7-O4	73.1(3)
O19-Gd3-O1	130.6(3)	O12-Gd7-O11	165.3(3)
O19-Gd3-O5	64.8(3)	O12-Gd7-O35	89.3(3)
O19-Gd3-O23	99.9(3)	O12-Gd7-N23	103.6(4)
O19-Gd3-N7	87.1(3)	O35-Gd7-N23	65.2(3)

Table S3. The results of coordination geometric configurations evaluated by SHAPE software for seven-coordinated Gd of complex **1**.

Label	Symmetry	Geometric configuration	Deviation parameters	
			Gd4/ Gd11/ Gd18	Gd7/ Gd14/ Gd21
HP-7	D_{7h}	Heptagon	30.083	29.939
HPY-7	C_{6v}	Hexagonal pyramid	23.138	22.557
PBPY-7	D_{5h}	Pentagonal bipyramid	1.790	1.786
COC-7	C_{3V}	Capped octahedron	6.817	7.051
CTPR-7	C_{2V}	Capped trigonal prism	5.126	5.191
JPBPY-7	D_{5h}	Johnson pentagonal bipyramid J13	4.821	4.724
JETPY-7	C_{3v}	Johnson elongated triangular pyramid J7	20.196	20.232

Table S4. The results of coordination geometric configurations evaluated by SHAPE software for eight-coordinated Gd of complex **1**.

Label	Symmetry	Geometric configuration	Deviation parameters		
			Gd1/ Gd8/ Gd15	Gd3/ Gd10/ Gd17	Gd6/ Gd13/ Gd20
OP-8	D_{8h}	Octagon	24.291	29.216	29.763
HPY-8	C_{7v}	Heptagonal pyramid	23.533	21.322	21.308
HBPY-8	D_{6h}	Hexagonal bipyramid	15.298	13.95	13.85
CU-8	O_h	Cube	8.06	13.38	12.845
SAPR-8	D_{4d}	Square antiprism	1.557	4.307	4.746
TDD-8	D_{2d}	Triangular dodecahedron	1.637	2.021	2.205
JGBF-8	D_{2d}	Johnson gyrobifastigium J26	15.892	10.603	10.637
JETBPY-8	D_{3h}	Johnson elongated triangular bipyramid J14	26.08	28.05	27.386
JBTPR-8	C_{2v}	Biaugmented trigonal prism J50	3.438	3.688	3.5
BTPR-8	C_{2v}	Biaugmented trigonal prism	2.762	2.858	2.647
JSD-8	D_{2d}	Snub diphenoid J84	4.648	3.209	3.206
TT-8	T_d	Triakis tetrahedron	8.771	14.161	13.612
ETBPY-8	D_{3h}	Elongated trigonal bipyramid	22.355	23.536	23.367

Table S5. The results of coordination geometric configurations evaluated by SHAPE software for nine-coordinated Gd of complex **1**.

Label	Symmetry	Geometric configuration	Deviation parameters	
			Gd2/ Gd9/ Gd16	Gd5/ Gd12/ Gd19
EP-9	D_{9h}	Enneagon	34.845	34.29
OPY-9	C_{8v}	Octagonal pyramid	19.725	19.883
HBPY-9	D_{7h}	Heptagonal bipyramid	15.388	14.644
JTC-9	C_{3v}	Johnson triangular cupola J3	16.003	15.539
JCCU-9	C_{4v}	Capped cube J8	10.032	10.077
CCU-9	C_{4v}	Spherical-relaxed capped cube	8.328	8.205
JCSAPR-9	C_{4v}	Capped square antiprism J10	3.022	3.259
CSAPR-9	C_{4v}	Spherical capped square antiprism	1.813	1.907
JTCTPR-9	D_{3h}	Tricapped trigonal prism J51	4.779	5.11
TCTPR-9	D_{3h}	Spherical tricapped trigonal prism	1.522	1.675
JTDIC-9	C_{3v}	Tridiminished icosahedron J63	11.697	12.214
HH-9	C_{2v}	Hula-hoop	11.282	10.745
MFF-9	C_s	Muffin	2.136	2.131

Table S6. Selected bond distances (Å) and bond angles (°) for complex **2**.

Gd1-O25 (μ_3-OH$^-$)	2.320(7)	Gd5-O22 (μ_3-OH$^-$)	2.292(8)
Gd1-O25A (μ_3-OH$^-$)	2.369(8)	Gd5-O22B (μ_3-OH$^-$)	2.391(7)
Gd1-O1 (μ_2-phO$^-$)	2.340(8)	Gd5-O4 (μ_2-phO$^-$)	2.329(9)
Gd1-O1B (μ_2-phO$^-$)	2.403(9)	Gd5-O4A (μ_2-phO$^-$)	2.426(9)
Gd1-O24 (μ_4-O^{2-})	2.484(15)	Gd5-O20 (μ_4-O^{2-})	2.5462(15)
Gd1-O2 (acyl oxygen)	2.439(8)	Gd5-O5 (acyl oxygen)	2.452(8)
Gd1-O7 (nitrate)	2.5316(14)	Gd5-O10 (nitrate)	2.511(14)
Gd1-O8 (nitrate)	2.579(11)	Gd5-O11 (nitrate)	2.566(16)
Gd1-N1	2.587(19)	Gd5-N5	2.601(11)
Gd1-Gd1A	3.5669(11)	Gd5---Gd5A	3.5828(12)
		Gd5---Gd4	3.882(1)
Gd2-O23 (μ_3-OH$^-$)×4	2.434(9)	Gd4-O21 (μ_3-OH$^-$)×4	2.454(8)
Gd2-O25 (μ_3-OH$^-$)×4	2.494(7)	Gd4-O22 (μ_3-OH$^-$)×4	2.511(8)
Gd2-O19 (μ_6-O^{2-})	2.617(13)	Gd4-O19 (μ_6-O^{2-})	2.716(13)
Gd2---Gd1	3.873(1)	Gd4---Gd3	3.6500(10)
Gd3-O21 (μ_3-OH$^-$)	2.335(8)	Gd3-O14 (nitrate)	2.475(11)
Gd3-O21B (μ_3-OH$^-$)	2.383(7)	Gd3-O13 (nitrate)	2.551(9)
Gd3-O23 (μ_3-OH$^-$)	2.335(8)	Gd3-O17 (nitrate)	2.485(11)
Gd3-O23A (μ_3-OH$^-$)	2.391(8)	Gd3-O16 (nitrate)	2.555(9)
Gd3-O19 (μ_6-O^{2-})	2.4822(8)	Gd3---Gd3A	3.5099(11)
Gd2---Gd3	3.6355(10)		
Gd1-O1-Gd1A	97.5(3)	Gd5-O4-Gd5B	97.8(3)
Gd2-O19-Gd3	90.9(3)	Gd3-O19-Gd3A	89.983(10)
Gd2-O19-Gd4	180.0	Gd3-O19-Gd3C	178.2(6)
Gd3-O19-Gd4	89.1(3)	Gd1-O25-Gd1B	99.0(3)
Gd1-O25-Gd2	107.1(3)	Gd1-O25-Gd2B	105.6(3)
Gd2-O23-Gd3	99.3(3)	Gd2-O23-Gd3B	97.8(3)
Gd3-O23-Gd3B	95.9(3)	Gd5-O22-Gd5A	99.8(2)
Gd4-O22-Gd5	107.8(4)	Gd4-O22-Gd5A	104.7(3)
Gd3-O21-Gd3A	96.1(3)	Gd3-O21-Gd4	99.3(3)
Gd3A-O21-Gd4	98.0(3)		
Gd1-O24-Gd1C	170.1(6)	Gd5-O20-Gd5C	168.5(5)
Gd1-O24-Gd1A	89.57(5)	Gd5-O20-Gd5A	89.43(5)

Symmetry codes: A y, -x+1/2, z; B -y+1/2, x, z; C -x+1/2, -y+1/2, z.

Table S7. The results of coordination geometric configurations evaluated by SHAPE software for nine-coordinated Gd of complex **2**.

Label	Symmetry	Geometric configuration	Deviation parameters				
			Gd1	Gd2	Gd3	Gd4	Gd5
EP-9	D_{9h}	Enneagon	31.039	36.92	30.122	36.567	30.963
OPY-9	C_{8v}	Octagonal pyramid	21.347	23.495	22.334	23.443	20.693
HBPY-9	D_{7h}	Heptagonal bipyramid	16.51	20.785	18.68	20.74	16.975
JTC-9	C_{3v}	Johnson triangular cupola J3	11.838	17.003	13.64	16.85	11.913
JCCU-9	C_{4v}	Capped cube J8	5.597	9.803	7.653	9.338	6.049
CCU-9	C_{4v}	Spherical-relaxed capped cube	5.015	9.476	6.933	9.324	5.493
JCSAPR-9	C_{4v}	Capped square antiprism J10	2.621	0.617	2.571	0.456	2.41
CSAPR-9	C_{4v}	Spherical capped square antiprism	2.164	0.326	1.905	0.444	1.96
JTCTPR-9	D_{3h}	Tricapped trigonal prism J51	3.354	2.709	2.491	2.579	3.481
TCTPR-9	D_{3h}	Spherical tricapped trigonal prism	3.552	1.626	2.642	1.768	3.356
JTDIC-9	C_{3v}	Tridiminished icosahedron J63	13.365	14.426	13.842	14.522	13.466
HH-9	C_{2v}	Hula-hoop	9.575	13.059	11.579	13.146	10.582
MFF-9	C_s	Muffin	2.419	1.213	1.859	1.334	2.157

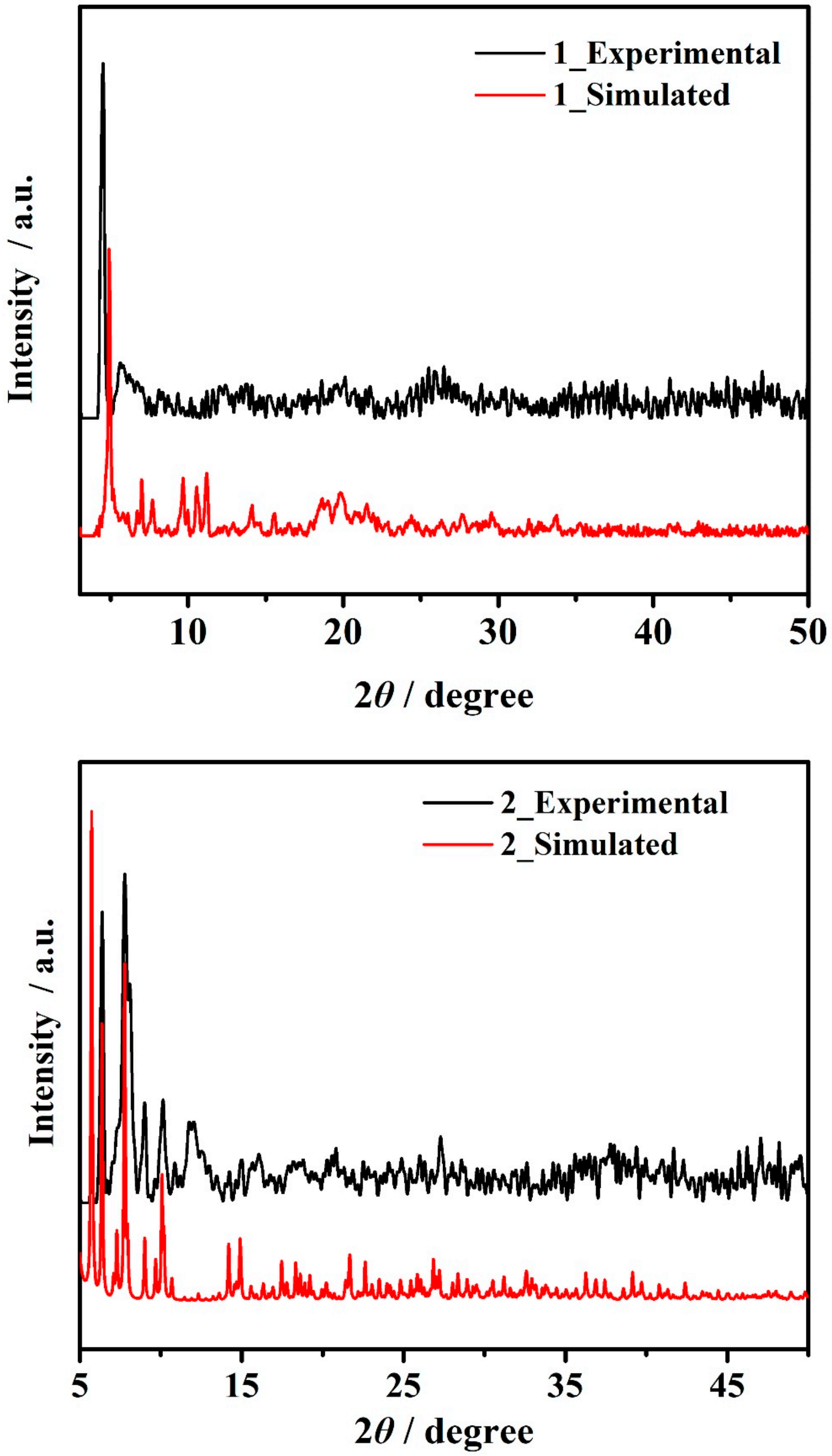

Figure S1. Powder diffraction pattern of complexes **1** and **2**.

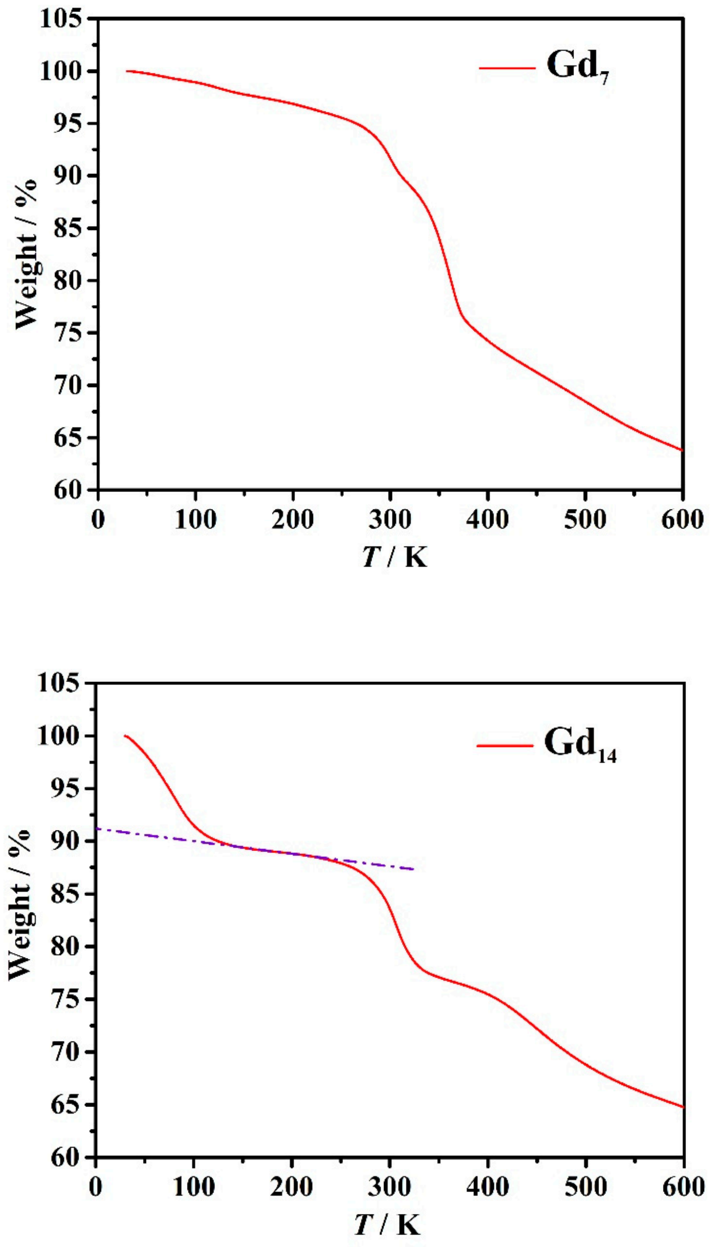

Figure S2. Thermogravimetric analysis of complexes **1** and **2**.

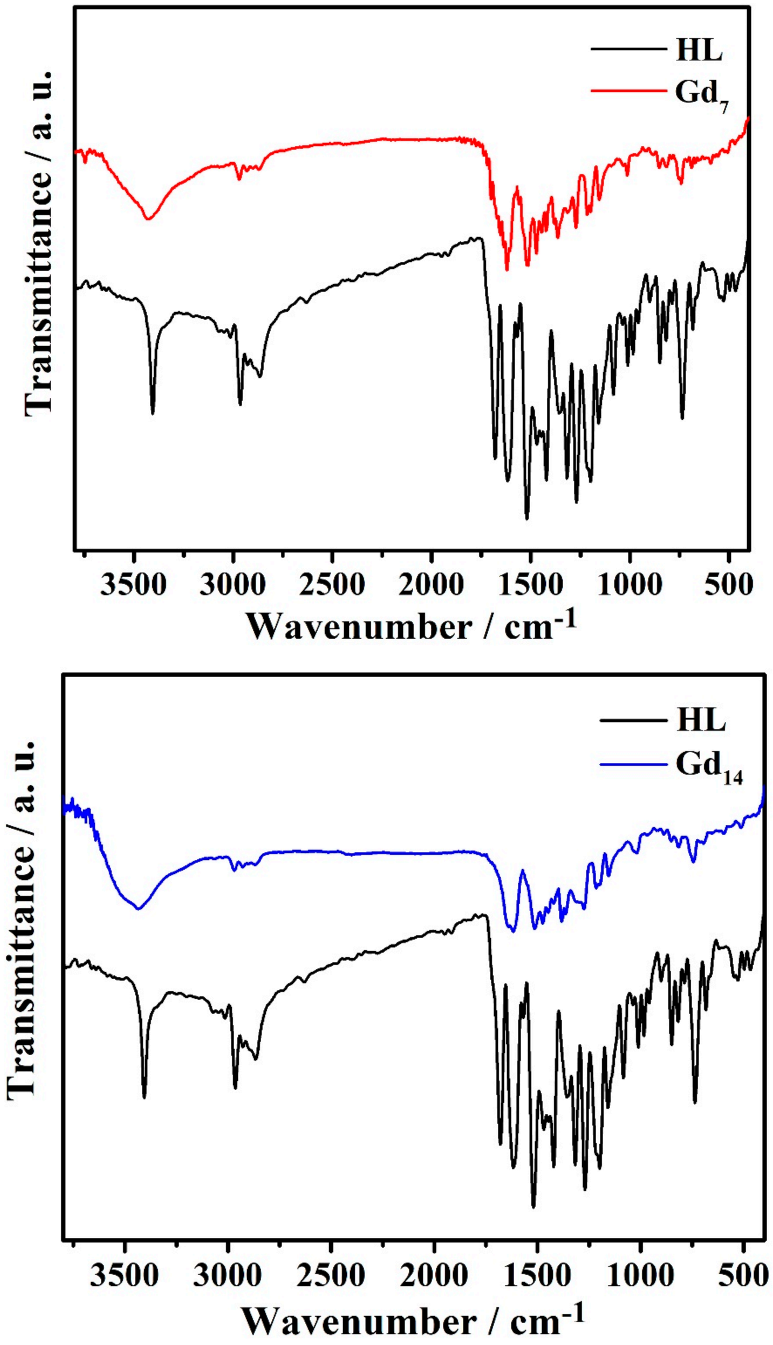

Figure S3. IR spectra of HL, complexes **1** and **2**.

(a)

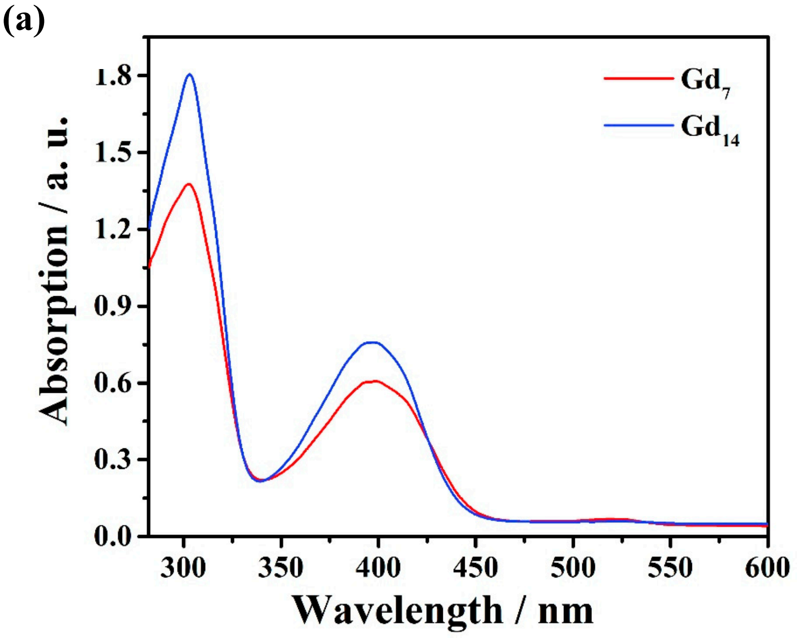

(b)

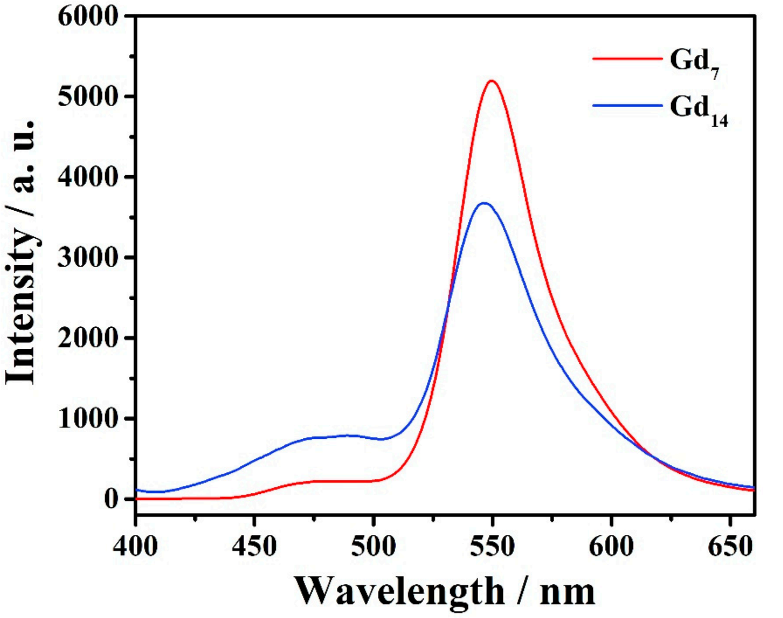

Figure S4. UV-vis (a) and fluorescence spectra (λ_{ex} = 354 nm, b) for complexes Gd_7 (1) and Gd_{14} (2) in MeOH (10 μM).

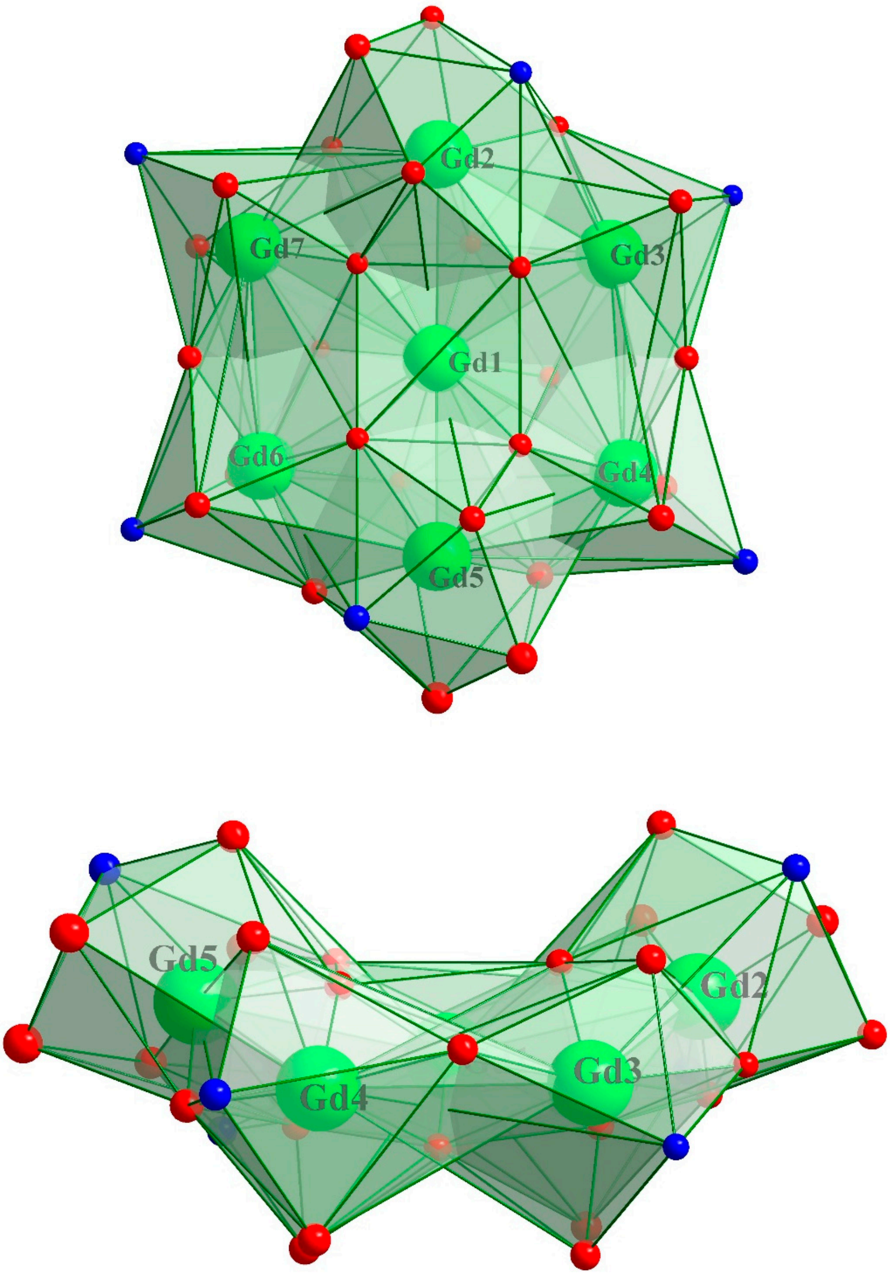

Figure S5. Top and side view of the heptanuclear surrounding polyhedral structures for complex **1**.

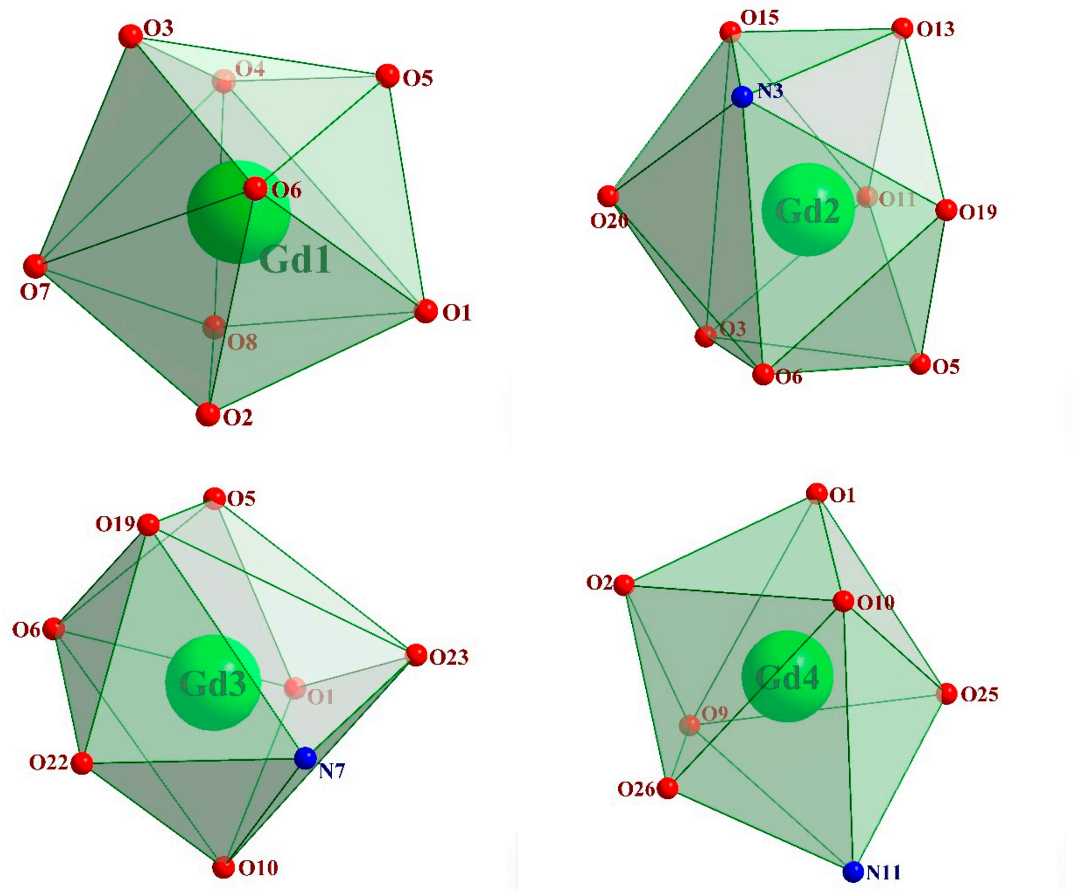

Figure S6. The coordinate lanthanide Gd^{III} surrounding polyhedral structures for complex **1**.

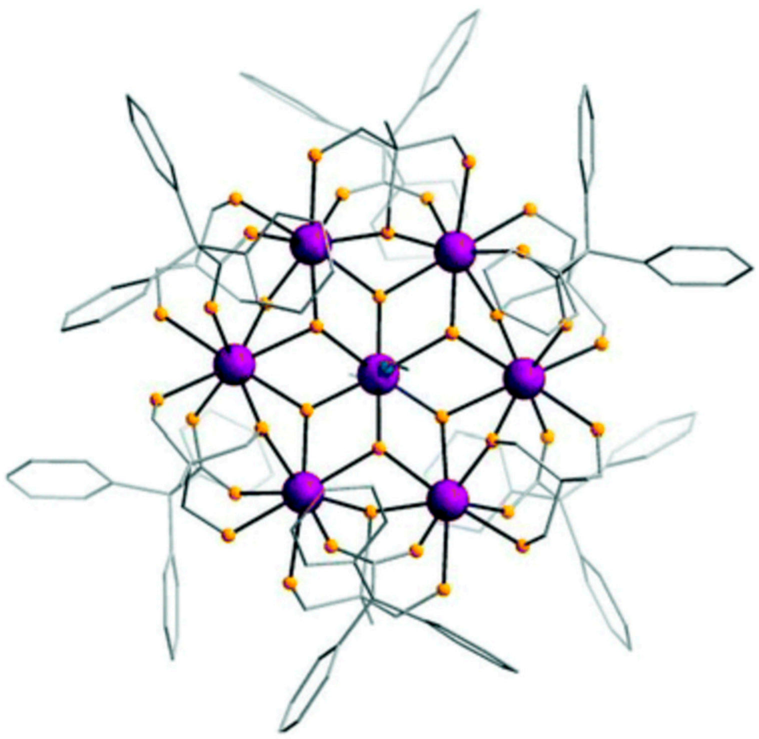

Figure S7. Structure of $[Gd_7(OH)_6(thmeH_2)_5(thmeH)(tpa)_6(MeCN)_2]^{2+}$ (Gd (purple), O (yellow), N (blue), C (skeletal). H atoms are not shown).
Chem. Commun. **2011***, 47*, 7650-7652. https://doi.org/10.1039/c1cc12252e.

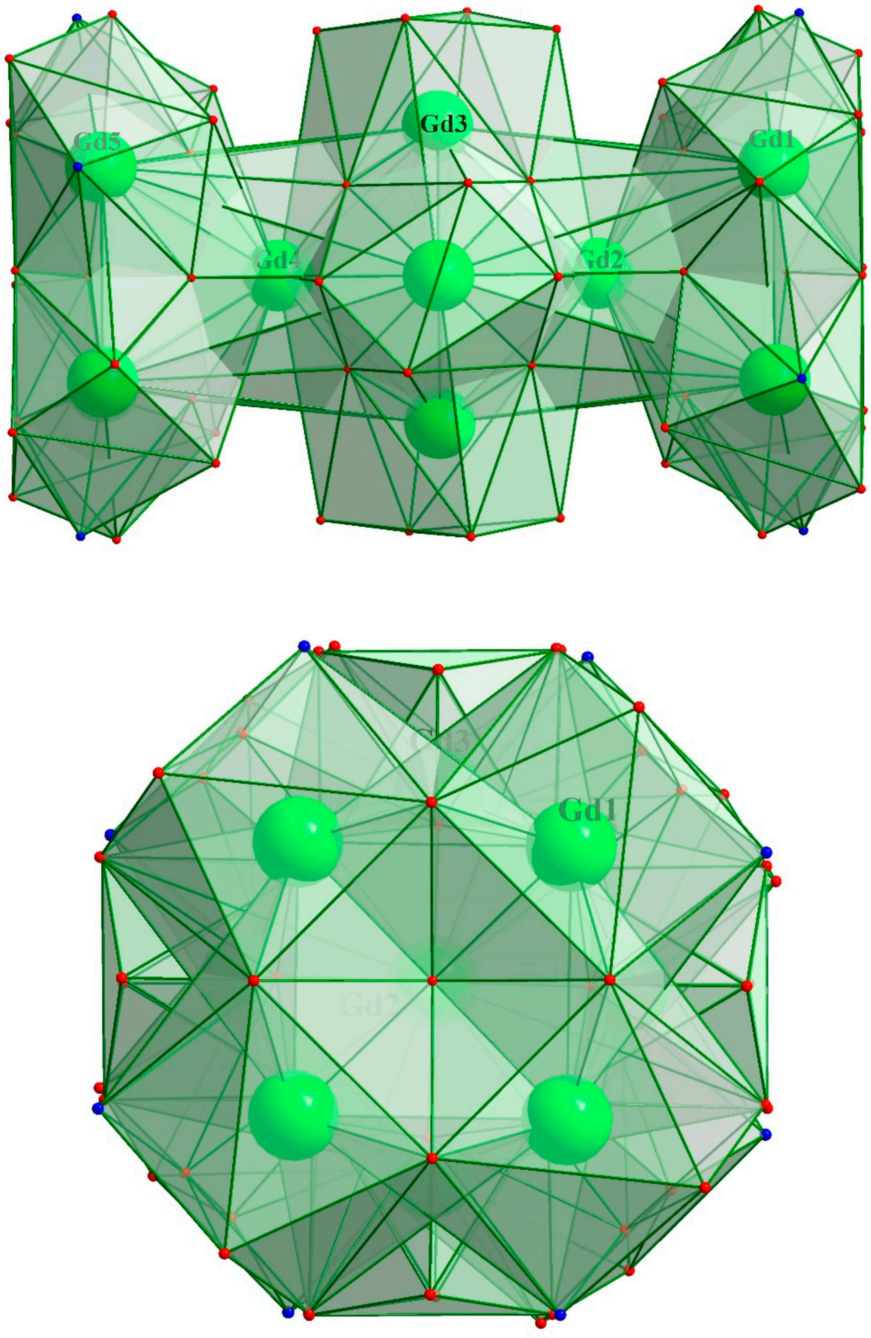

Figure S8. Drawings of the tetradecanuclear surrounding polyhedral structures for complex **2**.

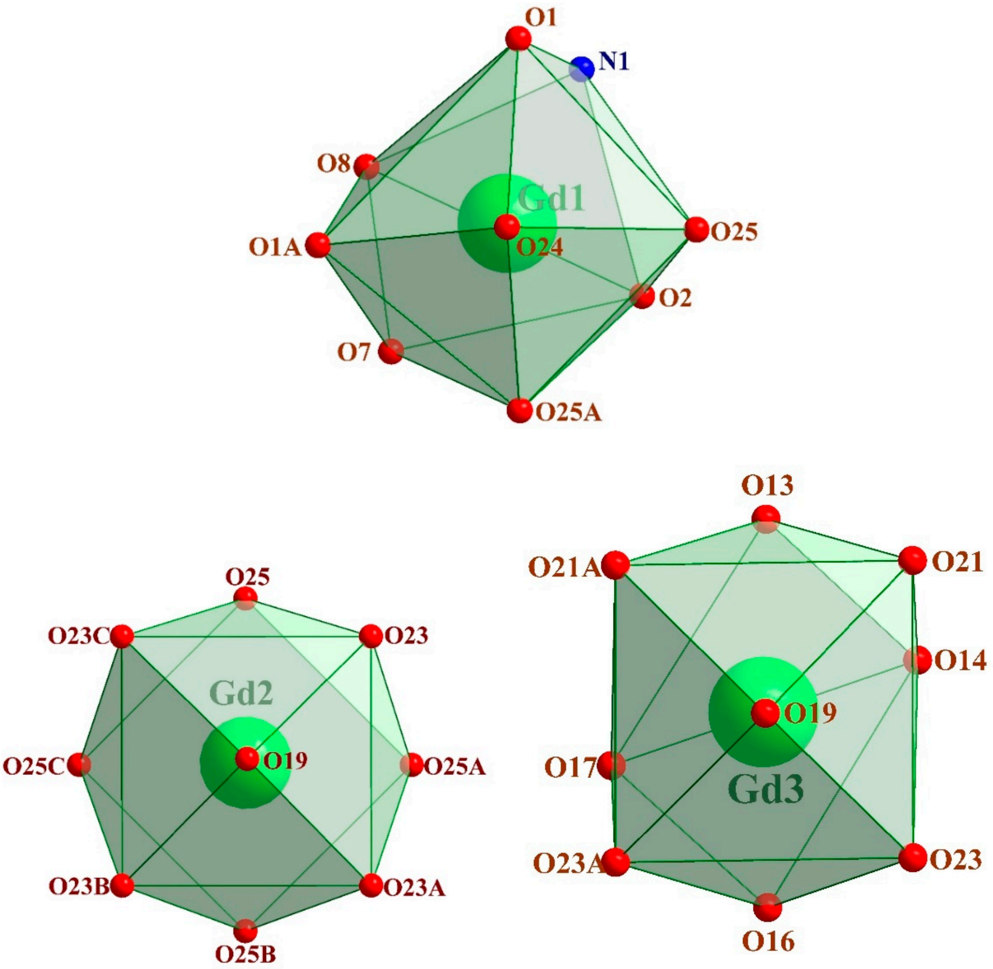

Figure S9. Drawings of the coordinate lanthanide Gd^{III} surrounding polyhedral structures for complex **2**.

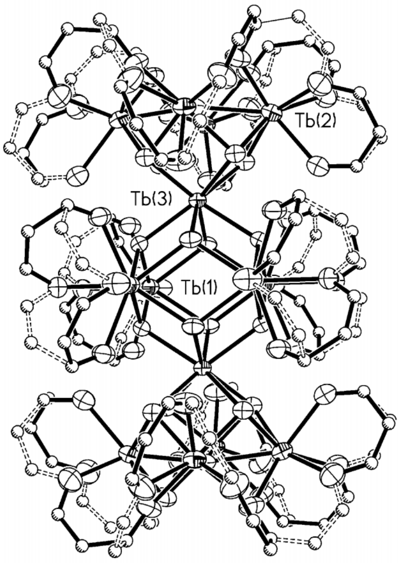

Figure S10. The molecular structure of $Ln_{14}(\mu_4\text{-}OH)_2(\mu_3\text{-}OH)_{16}(\mu\text{-}\eta^2\text{-}acac)_8(\eta^2\text{-}acac)_{16}$ (Ln = Tb and Eu, $acac^-$ = acetylacetonato).
Chem. Commun. **2002**, 368-369. https://doi.org/10.1039/b105969f.

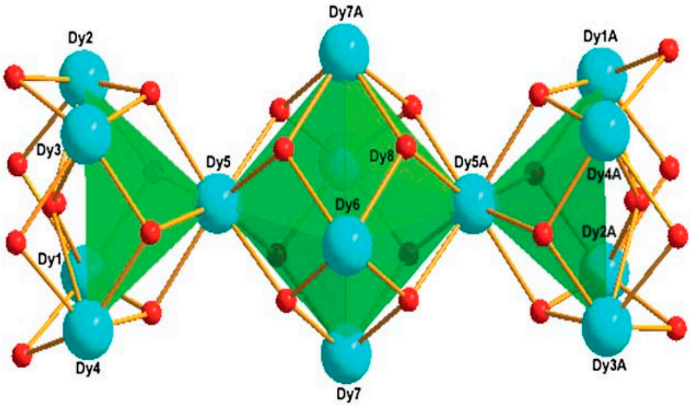

Figure S11. Polyhedral representation of the structure of $Ln_{14}(\mu_4\text{-}OH)_2(\mu_3\text{-}OH)_{16}(\mu\text{-}\eta^2\text{-}acac)_8(\eta^2\text{-}acac)_{16}\cdot 6H_2O$ (Ln = Dy and Tb) cluster core.
CrystEngComm **2011**, *13*, 3643-3645. https://doi.org/10.1039/c0ce00826e

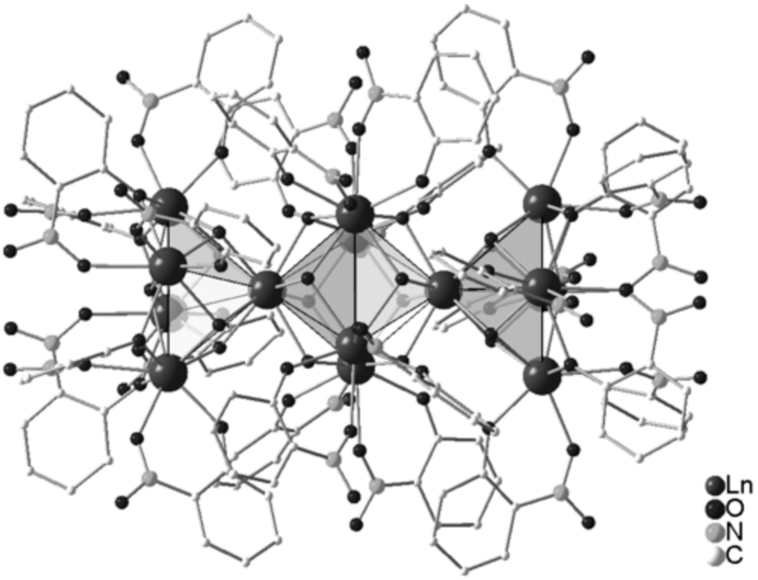

Figure S12. Solid-state structure of $H_{18}[Ln_{14}(\mu\text{-}\eta^2\text{-o-}O_2N\text{-}C_6H_4\text{-}O)_8(\eta^2\text{-o-}O_2N\text{-}C_6H_4\text{-}O)_{16}(\mu_4\text{-}O)2(\mu_3\text{-}O)_{16}]$ (Ln = Dy, Er, Tm, Yb； o-O_2N-C_6H_4-O = o-nitrophenolate) showing the atom labeling scheme, omitting hydrogen atoms.
J. Am. Chem. Soc. **2004***, 126*, 5213-5218. https://doi.org/10.1021/ja0396044.

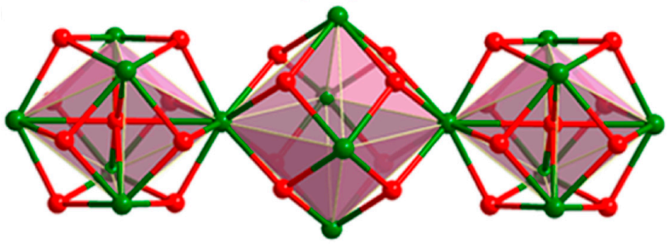

Figure S13. Polyhedron view of the $[Eu_{16}(tfac)_{20}(CH_3OH)_8(\mu_3\text{-}OH)_{24}(\mu_6\text{-}O)_2]$ cluster where ligands have been removed for clarity.
J. Am. Chem. Soc. 2022*, 144*, 5653-5660. https://doi.org/10.1021/jacs.2c01502.

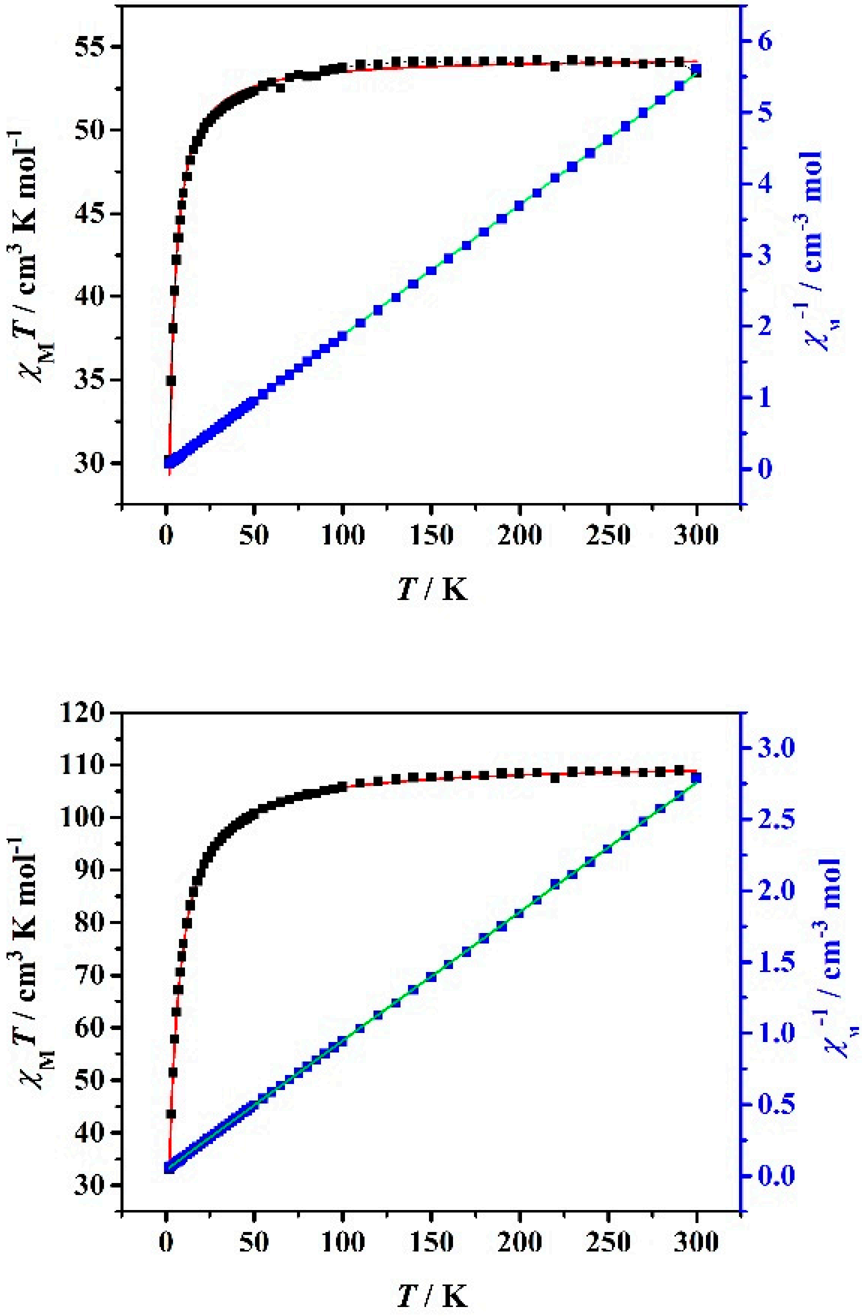

Figure S14. Temperature dependence of $\chi_M T$ for **1**(up) and **2**(bottom) under a 1000-Oe magnetic field in the range of 2–300 K. The solid curves represent the Curie-Weiss fit results.

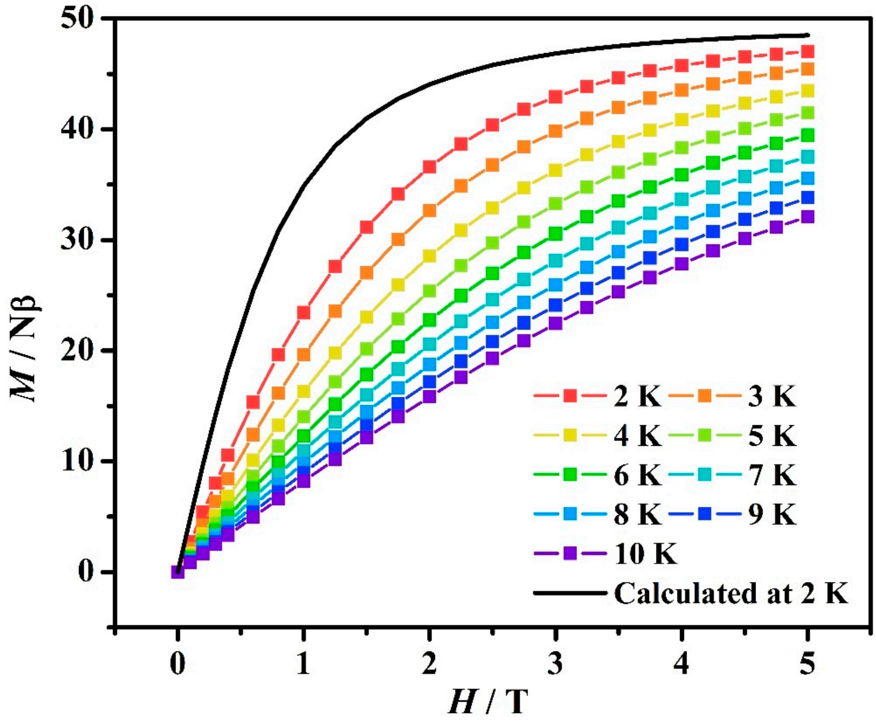

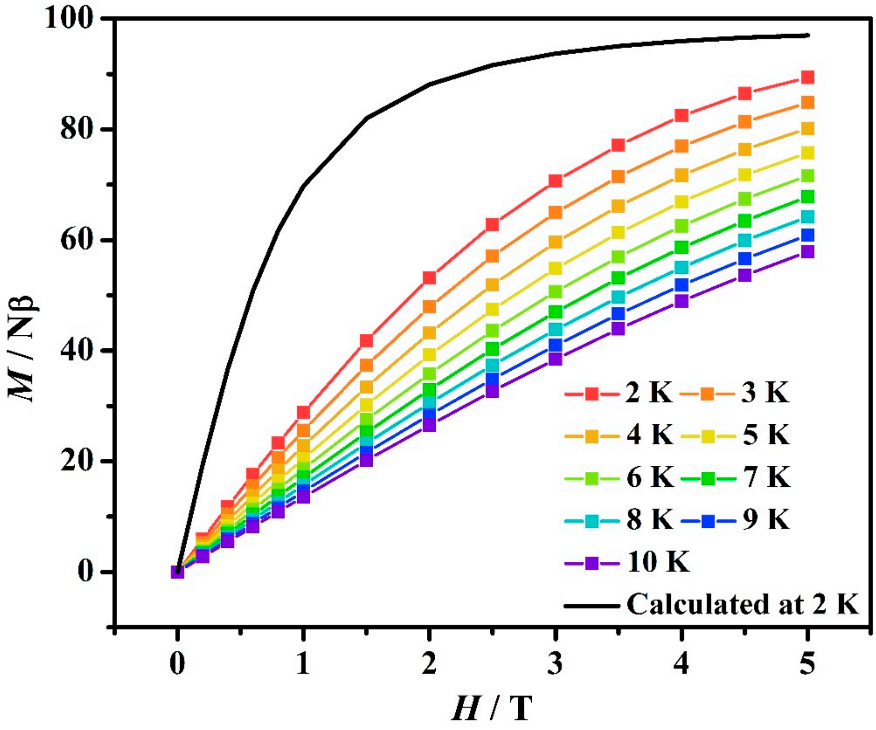

Figure S15. Field-dependence of the magnetization for **1**(up) and **2**(bottom) in the range of 2–10 K at 0 - 5 T. The solid curves represent the calculated Brillouin values for non-interacting S_{Gd} spins at 2 K.

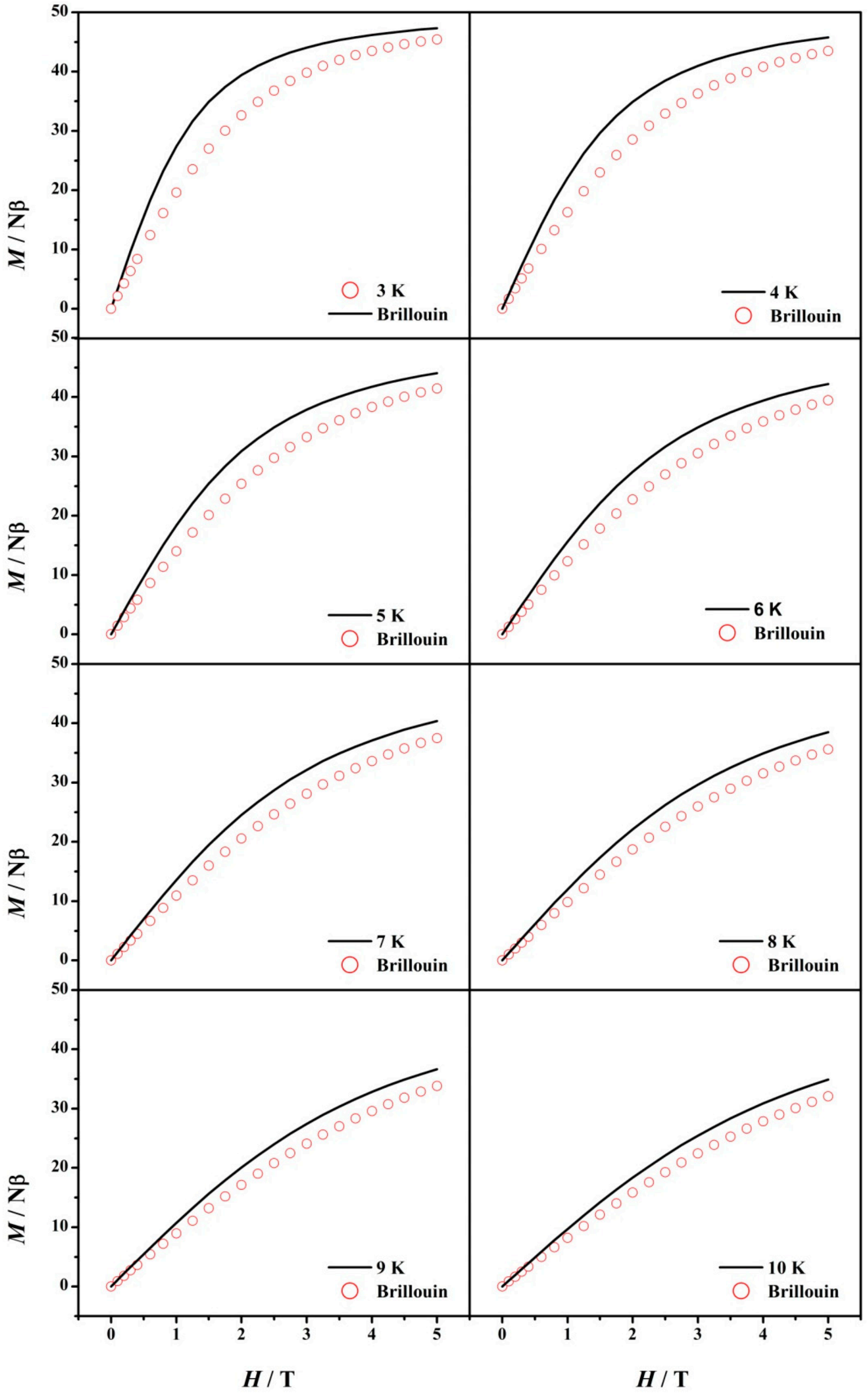

Figure S16. Field-dependence of the magnetization for **1** (Gd_7) in the range of 3–10 K. The solid curves represent the calculated Brillouin values for non-interacting S_{Gd} spins in the range of 3–10 K, respectively.

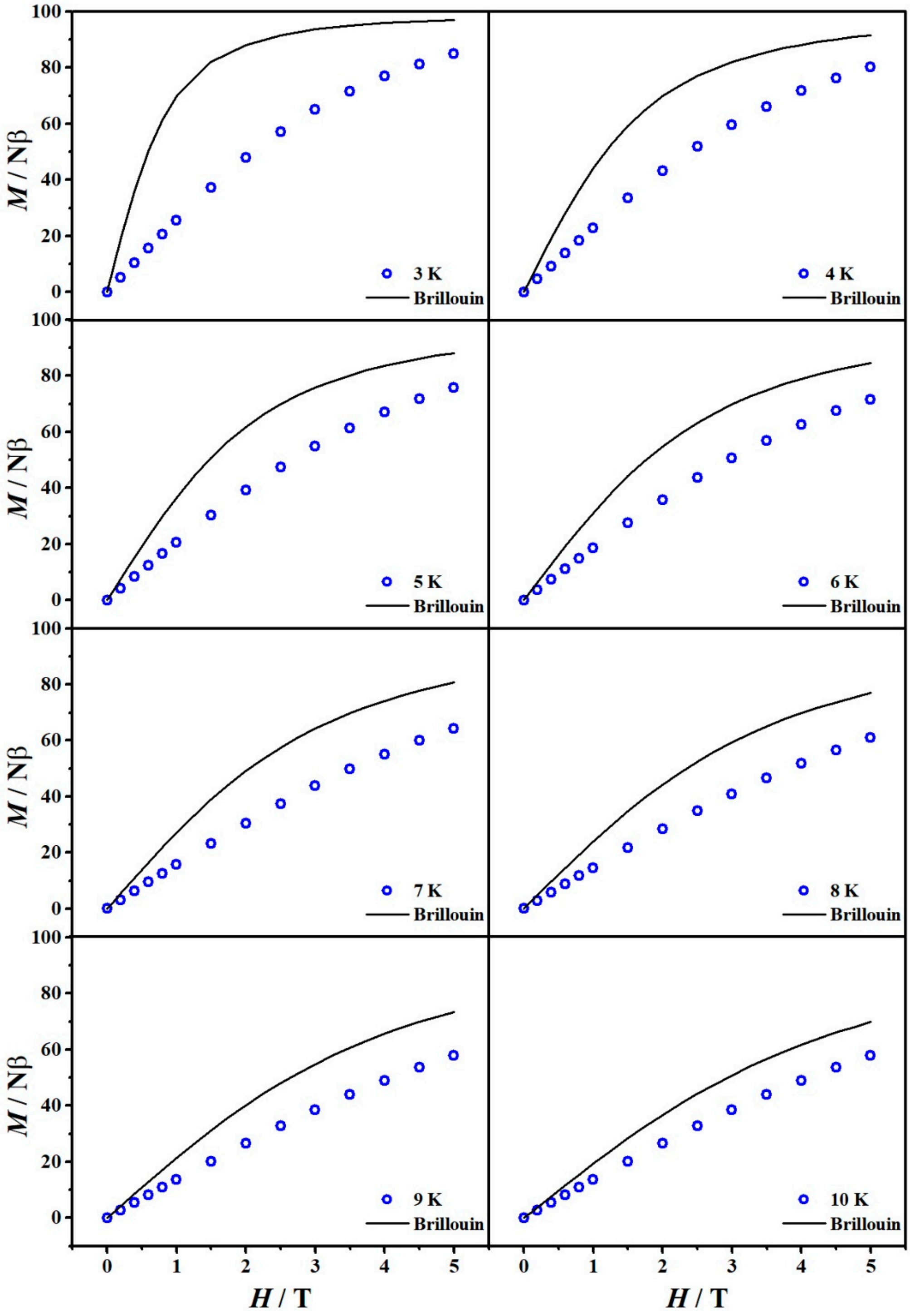

Figure S17. Field-dependence of the magnetization for **2** (Gd_{14}) in the range of 3–10 K at 0 - 5 T. The solid curves represent the calculated Brillouin values for non-interacting S_{Gd} spins in the range of 3–10 K, respectively.
